# MicroRNA in the Exosomes Mediated by Resveratrol to Activate Neuronal Cells

**DOI:** 10.3390/toxics12020122

**Published:** 2024-02-01

**Authors:** Zhendong Zhang, Qi Tao, Lixia Bai, Zhe Qin, Xiwang Liu, Shihong Li, Yajun Yang, Wenbo Ge, Jianyong Li

**Affiliations:** 1 Key Lab of New Animal Drug of Gansu Province, Key Lab of Veterinary Pharmaceutical Development of Ministry of Agriculture and Rural Affairs, Lanzhou Institute of Husbandry and Pharmaceutical Sciences of CAAS, Lanzhou 730050, China; 13027721013@163.com (Z.Z.); taoqi19951224@163.com (Q.T.); bailx552369@163.com (L.B.); qinzhe@caas.cn (Z.Q.); xiwangliu@126.com (X.L.); lzlishihong@163.com (S.L.); yangyue10224@163.com (Y.Y.); gewb1993@163.com (W.G.); 2 College of Life Sciences, South China Agricultural University, Guangzhou 510642, China

**Keywords:** resveratrol, Caco-2 cells, exosomes, miRNA, SH-SY5Y cells

## Abstract

Resveratrol (RSV), a polyphenol, is known to have a wide range of pharmacological properties in vitro. RSV may have therapeutic value for various neurodegenerative diseases via neuroprotective effects. However, it is not yet clear whether RSV can induce intestinal–brain interactions. It is assumed that the intestinal cells may secrete some factors after being stimulated by other substances. These secreted factors may activate nerve cells through gut–brain interaction, such as exosomes. In this study, it was discovered that Caco-2 cells treated with RSV secrete exosomes to activate SH-SY5Y neuronal cells. The results showed that secreted factors from RSV-treated Caco-2 cells activated SH-SY5Y. The exosomes of RSV-treated Caco-2 cells activated SH-SY5Y cells, which was manifested in the lengthening of the nerve filaments of SH-SY5Y cells. The exosomes were characterized using transmission electron microscopy and sequenced using the Illumina NovaSeq 6000 sequencer. The results showed that the miRNA expression profile of exosomes after RSV treatment changed, and twenty-six kinds of miRNAs were identified which expressed differentially between the control group and the RSV-treated group. Among them, three miRNAs were selected as candidate genes for inducing SH-SY5Y neural cell activation. Three miRNA mimics could activate SH-SY5Y neurons. These results suggested that the miRNA in intestinal exocrine cells treated with RSV may play an important role in the activation of SH-SY5Y neurons.

## 1. Introduction

Resveratrol (RSV) is an active polyphenol substance that is found in grapes and knotweed [1,2,3,4]. Studies showed that Resveratrol had anti-inflammatory, antioxidant, immunomodulatory, anticancer, neuroprotective, and cardioprotective properties [3,5,6,7,8]. There was a correlation between RSV levels and neuronal function [9]. RSV could protect the central nervous system of diabetic rats induced by streptozotocin and had a neuroprotective effect on the toxicity of amyloid β-induced hippocampal neurons in rats [10,11]. RSV could also prevent neurotoxicity caused by kainic acid and global cerebral ischemia damage in gerbils [12,13]. It has also been reported that RSV can exert its neuroprotective effect through increased heme oxygenase activity [14].

Extracellular vesicles are all membrane-bound vesicles released from cells into extracellular space. Extracellular vesicles can be divided into microbubbles (100~1000 nm) and exosomes (30~150 nm) according to their diameters. Exosomes are composed of lipid bilayer vesicles, which contain a lot of DNA, mRNA, miRNA, protein, and other genetic materials. The substances in exosomes can play an important role in cell proliferation and growth. Some studies showed that miRNA encapsulated in the exocrine body can be transferred to the target cells, thereby transferring miRNA signals from their parent cells. MiRNAs in the exosomes released by damaged podocytes can promote apoptosis of renal tubular epithelial cells. MiRNA is a 20~25 nucleotides-long noncoding regulatory RNA that is internally expressed and plays a major role as a regulator of gene expression. They can act as silencers of gene expression at the post-transcriptional level by inhibiting mRNA translation or mRNA degradation. In addition, miRNA can destabilize mRNA transcripts by binding to 5′ or 3′ untranslated regions. Therefore, miRNA plays a role as a promoter of normal basic biological processes, such as cell differentiation, proliferation, apoptosis, and survival by manipulating target genes.

Neurogenesis plays a vital role in maintaining neuronal quantity and viability through a process encompassing neuronal regeneration, differentiation, and synapse formation. The production and release of neurotrophic factors, such as nerve growth factor (NGF), brain-derived neurotrophic factor (BDNF), and glial cell line-derived neurotrophic factor (GDNF), serve as significant catalysts for neurogenesis, exerting regulatory control over neuronal growth, survival, and differentiation. The Caco-2 cell model (Caco-2), a human clone of colorectal adenocarcinoma cells, is used to predict the constituent’s intestinal absorption. This cell line is a useful research tool that can reveal that drugs or extracellular vesicles stimulate further differentiation of nerve cells. Human SH-SY5Y cells are commonly employed in vitro to simulate both undifferentiated and differentiated neuronal cells. These cells exhibit the presence of dopaminergic markers, such as DAT and tyrosine-hydroxylase (TH), and when subjected to various stimuli they demonstrate a mature neuron-like phenotype characterized by a spindle-shaped morphology, extensive neurite outgrowth, and heightened expression of TH and neuronal markers (e.g., neuron-specific enolase, synaptophysin, and synaptic-associated protein-97). In this study, it was investigated whether RSV-mediated extracellular vesicles produced by Caco-2 cells can activate neural cells. At the same time, second-generation sequencing technology was used to sequence miRNAs in extracellular vesicles to elucidate the molecular mechanism of gut-brain interactions induced by miRNAs.

## 2. Materials and Methods

### 2.1. Chemicals

Resveratrol (RSV) (purity ≥ 98%) and retinoic acid (RA) (purity ≥ 98%) were purchased from Sigma (St. Louis, MO, USA). TranswellTM cell culture dishes were obtained from Corning Costar Corp (Cambridge, MA, USA). Fetal bovine serum, MEM glucose medium, and cell culture flasks were from Gibco (Grand Island, NY, USA). Merck provided Neuro-Chrom Pan Neuronal Marker (Billerica, MA, USA). A MiRCURY exosome cell kit was purchased from Qiagen (Benelux B.V., Germany). The goat anti-rabbit IgG antibody Alexa Fluor 555 was purchased from Thermo Fisher Scientific, Inc. (Waltham, MA, USA). Exosome inhibitors (GW4869) were obtained from MedChemExpress (Monmouth Junction, NJ, USA). Anti-CD9 antibody (ab286172), Anti-CD63 antibody (ab275273), Anti-CD81 antibody (ab286173), Anti-NEFM antibody (ab7794), and Anti-BDNF antibody (ab108319) were obtained from Abcam (Cambridge, MA, USA).

### 2.2. Cell Culture

Caco-2 cells were maintained in media containing 20% fetal bovine serum (FBS), 1% glutamax, and 1% sodium pyruvate at 37 °C under humidified atmospheric conditions containing 5% CO_2_. SH-SY5Y cells were cultured in this medium at a 10% dilution for 24 h. An exosome collection kit was used to isolate exosomes from RSV-treated Caco-2 cells. A BCA protein assay kit was used to quantify exosomes. A protein equivalent to 90 ng of exosomes was added to SH-SY5Y cells for 24 h.

### 2.3. Cell Viability

Cell Counting Kit-8 (CCK-8) was used to detect the viability of Caco-2 cells. The CCK-8 cell viability detection kit contains WST-8 (2-(2-methoxy-4-nitrophenyl)-3-(4-nitrophenyl)-5-(2,4-disulfobenzene)-2H-tetrazole monosodium salt). In the presence of an electronic carrier, WST-8 is oxidized and reduced by intracellular dehydrogenase to produce water-soluble–orange-yellow methane dye, which can be dissolved in a tissue culture medium. The amount of methane generated is proportional to the number of living cells.

### 2.4. Measurement of Neurite Growth on SH-SY5Y Cells

The method for measuring the axonal growth of SH-SY5Y cells was carried out according to our previous experimental steps [15]. In brief, 2 × 10^4^ SH-SY5Y cells were seeded in 6-well plates. After SH-SY5Y cells were treated with culture medium or exosomes, the cells were fixed with 4% paraformaldehyde for 15 min and then blocked with blocking buffer for 1 h. The SH-SY5Y cells were incubated with Neuro-Chrom Pan Neuronal Markerat for 24 h. After washing three times with PBS, the SH-SY5Y cells were stained with Alexa Fluor 555 goat anti-rabbit IgG antibody at 25 °C for 1 h. The SH-SY5Y cells were further stained with Hoechst 33342 for 10 min and the staining of the cells was observed under a confocal laser scanning microscope (LSM 800, Zeiss, Germany). For quantifying the number of neurites per cell, neurite-bearing cells were counted from at least three randomly selected microscopic fields with an average of 40 cells per field.

Additionally, neurite length was measured from all neurite-bearing cells and the length of each neurite was quantified by using Image J 14 (Bethesda, MD, USA). Neurite lengths that were counted from three different images and at least 30 neurites per condition were quantified. Independent cell culture experiments were conducted in triplicate.

### 2.5. Protein Expression Analysis

The expressions of β-actin, neurofilament medium polypeptide (NEFM), and brain-derived neurotrophic factor (BDNF) were analyzed in SH-SY5Y cells via Western blot. The method for determining protein expression follows our previous experimental steps [15]. In brief, the collected total protein was separated using a precast SDS-PAGE gel. The separated target protein was transferred to a polyvinylidene fluoride membrane. The blots were incubated with the primary antibody for 24 h, and they were then incubated with the horseradish peroxidase-conjugated secondary antibody for 1 h. Protein bands were detected using a BIO-RAD imaging system (BIO-RAD, Hercules, CA, USA).

### 2.6. Establishment of Caco-2 Cells Model

In 12-well Transwell polycarbonate membranes, Caco-2 cells were seeded at 10^5^ per well. Every other day, the medium was changed. Every day, Caco-2 cells were observed for their morphology. When the resistance value of Caco-2 is greater than 1000 Ω, RSV as 128 μM or 256 μM was added to the apical Caco-2 cells.

### 2.7. Exosome Isolation

Exosomes were isolated with the miRCURY exosome cell kit (Qiagen, Benelux B.V., Germany) following the manufacturer’s instructions [16].

### 2.8. Transmission Electron Microscopy (TEM)

The TEM determination of exosomes separated with ultracentrifugation refers to our previous experimental steps [15]. In brief, one drop of resuspended exosomes was put on a copper mesh for 5 min. The fluid was then absorbed from the edges of the copper mesh with filter paper. The copper mesh was stained with 1% phosphotungstic acid, 44-hydrate for 1 min. The sample was dried for 20 min at room temperature after the staining solution was absorbed by the filter paper. The preparations were examined under a transmission electron microscope (FEI, Tecnai G2 Spirit BioTwin, Hillsboro, OR, USA) at an acceleration voltage of 80 kV.

### 2.9. RNA Sequencing (RNA-seq) and Bioinformatic Analysis

RNA was extracted using the RNeasy Micro Kit (Qiagen, Benelux B.V., Germany) following the guidelines provided by the manufacturer. To assess the quality of RNA, an Agilent 2100 Bioanalyzer was employed. To compare the control and experimental samples, we computed ratios based on the normalized signal intensities of every probe. TargetScan (http://www.targetscan.org/, accessed on 7 September 2021) was utilized to predict the target genes of miRNAs. We evaluated the pertinent functional classifications by utilizing the Gene Ontology (GO) and Kyoto Encyclopedia of Genes and Genomes (KEGG).

### 2.10. Preparation of miRNA Mimics

miR-199a-3p mimics, miR-215-5p mimics, miR-105-5p mimics, and the corresponding negative controls used were synthesized by GenePharma (Shanghai, China). The miRDB database contains the specific sequences of three miRNAs (https://mirdb.org/, accessed on 5 January 2022).

miR-199a-3p (ACAGUAGUCUGCACAUUGGUUA);

miR-215-5p (AUGACCUAUGAAUUGACAGAC);

miR-105-5p (UCAAAUGCUCAGACUCCUGUGGU).

MiRNA mimics were transfected into cells to enhance miRNA activity.

### 2.11. Statistical Analysis

The data are presented as means and standard deviations. Using one-way ANOVA and Duncan’s multiple comparisons, we analyzed the differences among treatment groups. Statistical significance was considered at *p* < 0.05.

## 3. Results

### 3.1. The Medium of Caco-2 Cells Treated with Resveratrol (RSV) Could Induce Neurite Outgrowth in SH-SY5Y Cells

**Figure 1:**
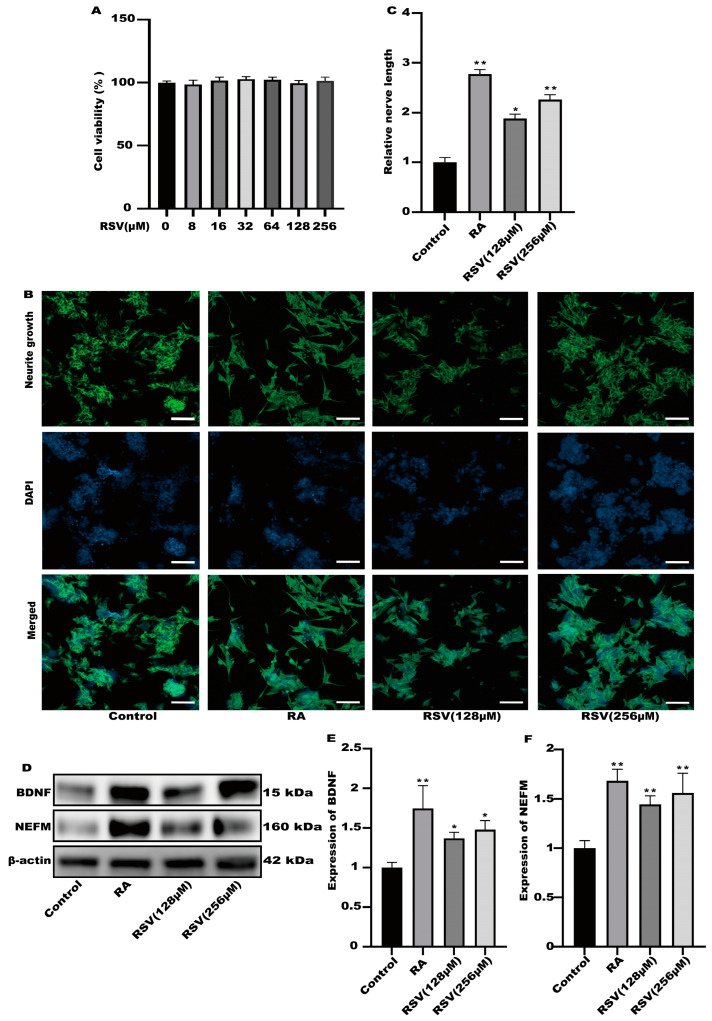
The culture medium of Caco-2 cells could activate SH-SY5Y cells. (**A**) The effects of different concentrations of RSV (8~256 μM) on Caco-2 cell viability. (**B**) Representative fluorescence images of SH-SY5Y were taken with a confocal laser microscope, scale bars: 100 µm. (**C**) Neurofilament medium polypeptide was quantitatively determined via ImageJ version 1.54. (**D**–**F**) The expression of brain-derived neurotrophic factor (BDNF) and neurofilament medium polypeptide (NEFM) was measured. Values are presented as the means ± SD where applicable (*n* = 6). Differences were considered significant when the *p* value was <0.05 (* *p* < 0.05, ** *p* < 0.01). The normal group without any treatment was treated as the control group. The RA group with retinoic acid treatment was treated as the positive group. The RSV (128 μM) group and RSV (256 μM) group were treated as the experimental groups.

To investigate the activation of neurons via RSV, we utilized RSV-treated Caco-2 cell culture media to stimulate neuronal cells and assess neurite growth. The results of cell viability indicated that the viability of Caco-2 cells remained unaffected by 256 μM RSV (Figure 1A). In the culture of SH-SY5Y cells, a 10% dilution of the RSV-treated Caco-2 cell medium was utilized. As a positive control, retinoic acid (RA) was utilized. According to Figure 1B,C, the Caco-2 cell culture medium exhibited a remarkable ability to enhance the growth of neurites in SH-SY5Y cells and significantly increased the expression levels of BDNF and NEFM in the RSV treatment group and the RA treatment group, when compared to the control group. BDNF promotes neuronal differentiation, growth, and survival (Figure 1D–F). Interestingly, Caco-2 cells induced via RSV may secrete factors that promote SH-SY5Y cell neurite growth.

### 3.2. Neurite Growth Was Induced in SH-SY5Y Cells by Factors Released from RSV-Treated Caco-2 Cells

**Figure 2:**
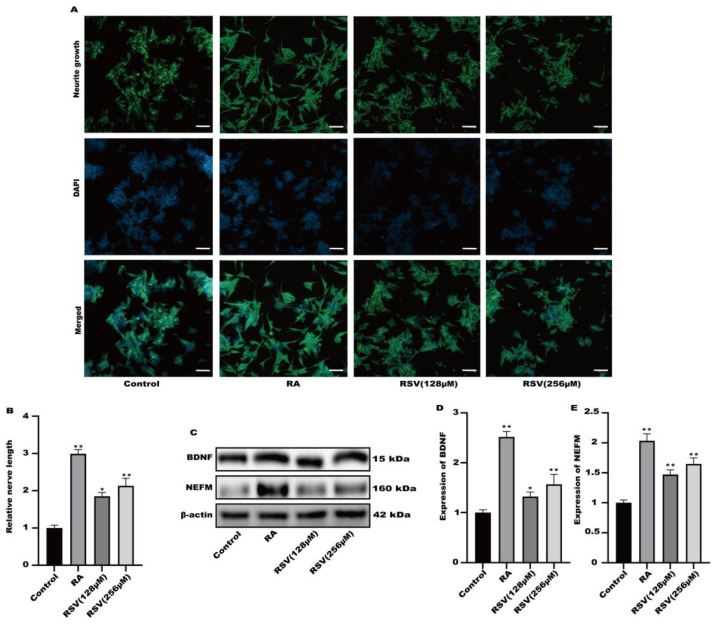
Activation of SH-SY5Y cells by factors released from Caco-2 cells cultured on permeable supports. (**A**) Representative fluorescence images of SH-SY5Y were taken with a confocal laser microscope, scale bars: 100 µm. (**B**) Neurofilament medium polypeptide was quantitatively determined via ImageJ version 1.54. (**C**–**E**) The expression of BDNF and NEFM was measured. Values are presented as the means ± SD where applicable (n = 6). Differences were considered significant when the *p* value was <0.05 (* *p* < 0.05, ** *p* < 0.01).

Factors released by Caco-2 cells treated with RSV stimulated the growth of neurites in SH-SY5Y cells. RSV was added to TranswellTM dishes containing cultured Caco-2 cells. Cytokines were gathered in a limited quantity of cell culture medium on the lower side. Figure 2A,B demonstrated that the application of RSV to the Caco-2 cell culture medium stimulated the development of neurites in SH-SY5Y cells. This medium also caused the induction of BDNF and NEFM in SH-SY5Y cells, as shown in Figure 2C–E. Secretory factors released by RSV-treated Caco-2 cells could potentially trigger nerve cell activation, facilitating intestinal–brain interactions caused by RSV.

### 3.3. SH-SY5Y Cell Neuritis Was Induced by Exosomes Produced from RSV-Treated Caco-2 Cells

**Figure 3:**
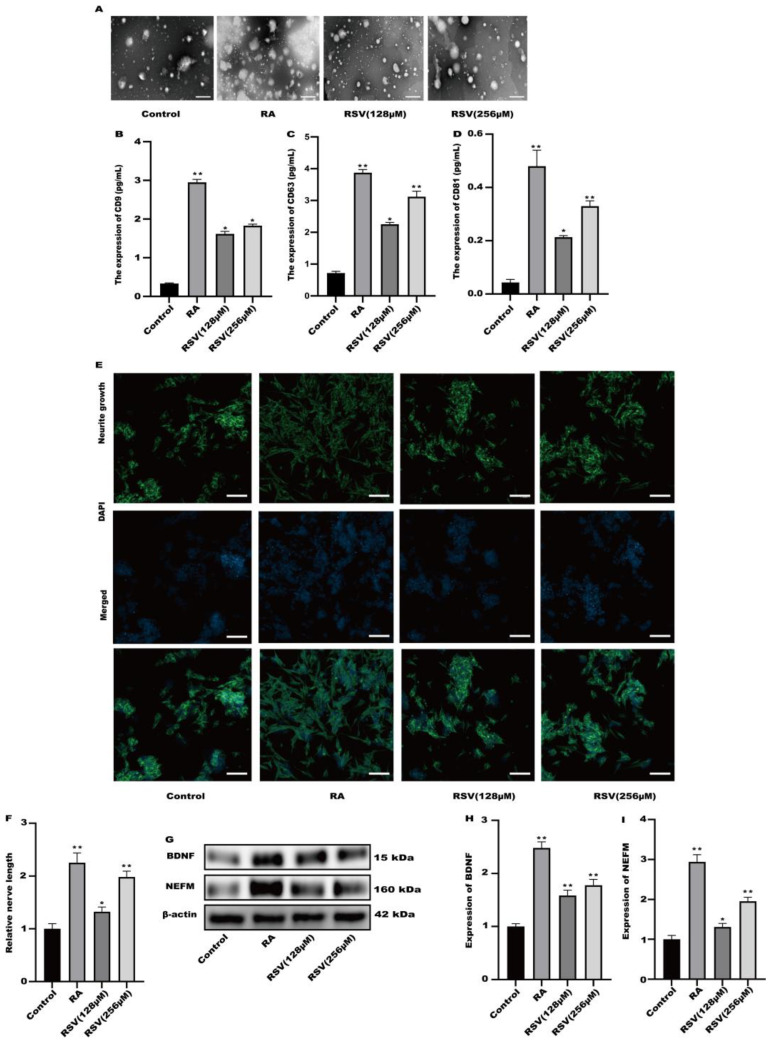
Activation of SH-SY5Y cells by exosomes derived from Caco-2 cells. (**A**) Exosomes were characterized via transmission electron microscopy, scale bars: 100 nm. (**B**–**D**) The concentration of CD9, CD63, and CD81 in the sample containing the purified exosomes was quantified with ELISA. (**E**) Representative fluorescence images of SH-SY5Y were taken with a confocal laser microscope, scale bars: 100 µm. (**F**) Neurofilament medium polypeptide was quantitatively determined via ImageJ version 1.54. (**G**–**I**) The expression of BDNF and NEFM was measured. Values are presented as the means ± SD where applicable (n = 6). Differences were considered significant when the *p* value was <0.05 (* *p* < 0.05, ** *p* < 0.01).

Exosomes produced from RSV-treated Caco-2 cells were isolated using a kit designed for exosome collection. Exosomes were characterized using transmission electron microscopy. Figure 3A illustrates the utilization of transmission electron microscopy for the characterization of every exosome group. To compare the exosome abundance, the concentrations of extracellular vesicle markers, namely CD9, CD63, and CD81, in the purified exosome sample were quantified using ELISA. Compared to the control, the amount of CD9, CD63, and CD81 markers increased in a concentration-dependent manner in the RSV-treated samples as well as in the RA-treated one as a positive control (Figure 3B–D). After being exposed to exosomes for a duration of 24 h, the findings from Figure 3E,F indicated a notable enhancement in neurite growth among SH-SY5Y cells when treated with 128 μM and 256 μM RSV. Compared to the control cells (Figure 3G–I), the exosomes stimulated the expression of BDNF and NEFM in SH-SY5Y cells. This study showed that exosomes generated by Caco-2 cells treated with RSV have a notable ability to enhance the growth of neurites in SH-SY5Y cells.

### 3.4. SH-SY5Y Cells Could Not Be Activated after the Production of Exosomes Was Inhibited

**Figure 4:**
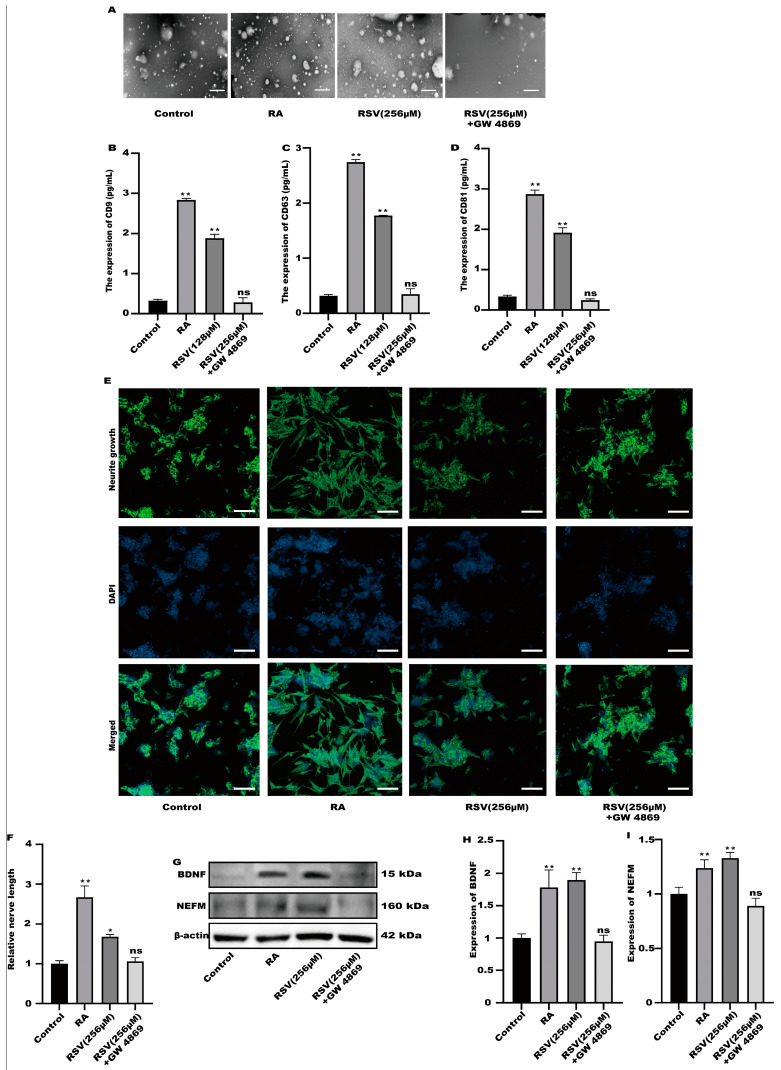
SH-SY5Y cells could not be activated after the production of exosomes was inhibited. (**A**) Exosomes were characterized with transmission electron microscopy, scale bars: 100 nm. (**B**–**D**) The concentrations of CD9, CD63, and CD81 in the sample containing the purified exosomes were quantified with ELISA. (**E**) Representative fluorescence images of SH-SY5Y were taken with a confocal laser microscope, scale bars: 100 µm. (**F**) Neurofilament medium polypeptide was quantitatively determined via ImageJ version 1.54. (**G**–**I**) The expression of BDNF and NEFM were measured. Values are presented as the means ± SD where applicable (n = 6). Differences were considered significant when the *p* value was <0.05 (* *p* < 0.05, ** *p* < 0.01). Compared to control; ns means no significant difference (*p* > 0.05).

GW4869 is an exosome inhibitor, which can inhibit the formation of exosomes. GW4869 was used to inhibit the production of exosomes. As shown in Figure 4A, GW4869 could significantly inhibit the production of extracellular vesicles by cells. To compare the exosome abundance, CD9, CD63, and CD81 in the purified exosome sample were quantified using ELISA. RSV treatment alone increased CD9, CD63, and CD81 concentrations; however, the addition of GW4869 with RSV decreased them to a negative control level (Figure 4B–D). The result showed that inhibitors can inhibit the production of exosomes. Furthermore, compared with the control group, there was no significant difference in the synaptic length of SH-SY5Y cells (Figure 4E,F), indicating that SH-SY5Y cells were not activated in the preparation group. Compared with the control group, the protein in the inhibitor group exhibited no significant difference (Figure 4G–I). The results showed that Caco-2 cells activated SH-SY5Y cells by producing exosomes.

### 3.5. Analysis of Exosome miRNA Expression Profiles

**Figure 5:**
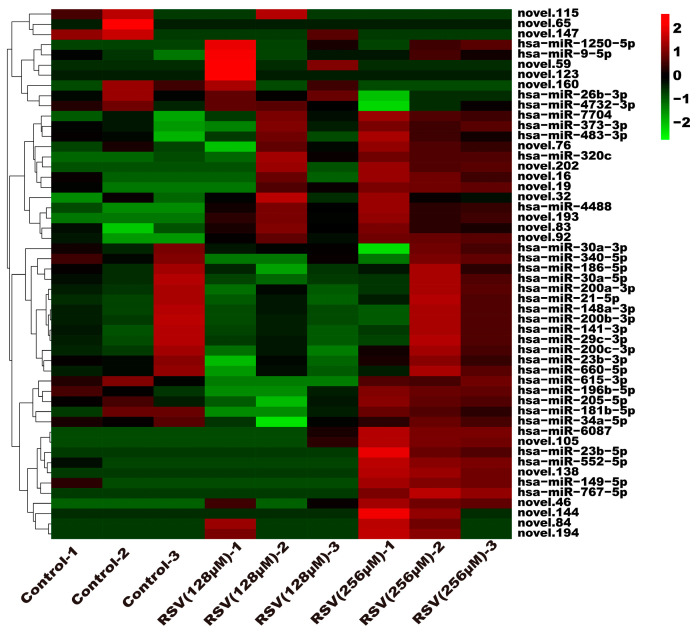
Expression profile analysis of exosome miRNAs.

**Figure 6:**
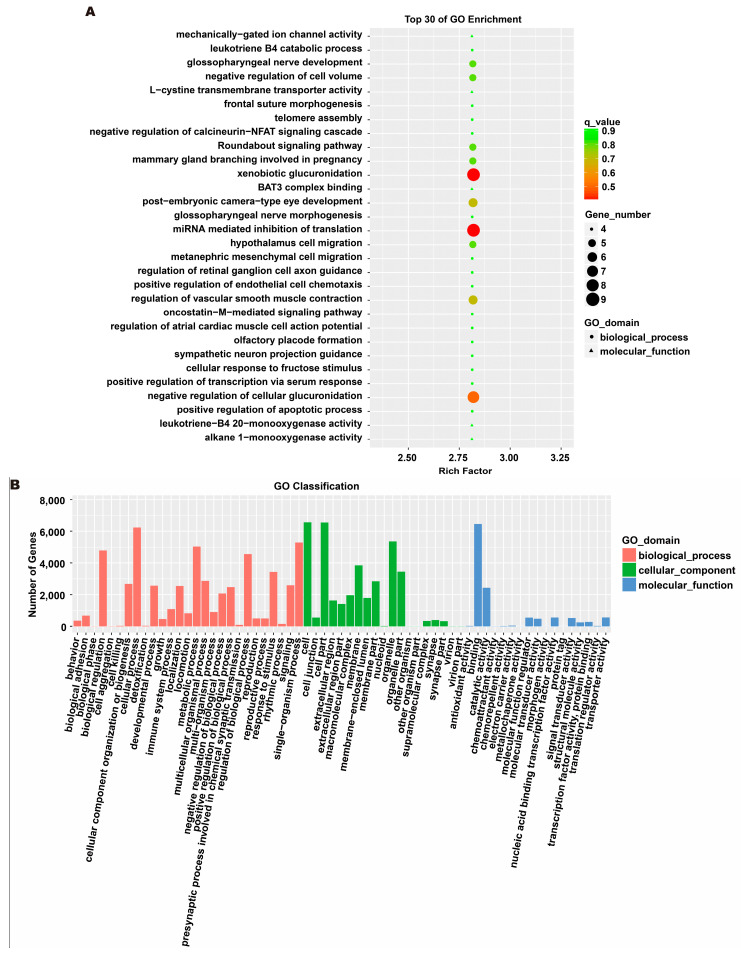

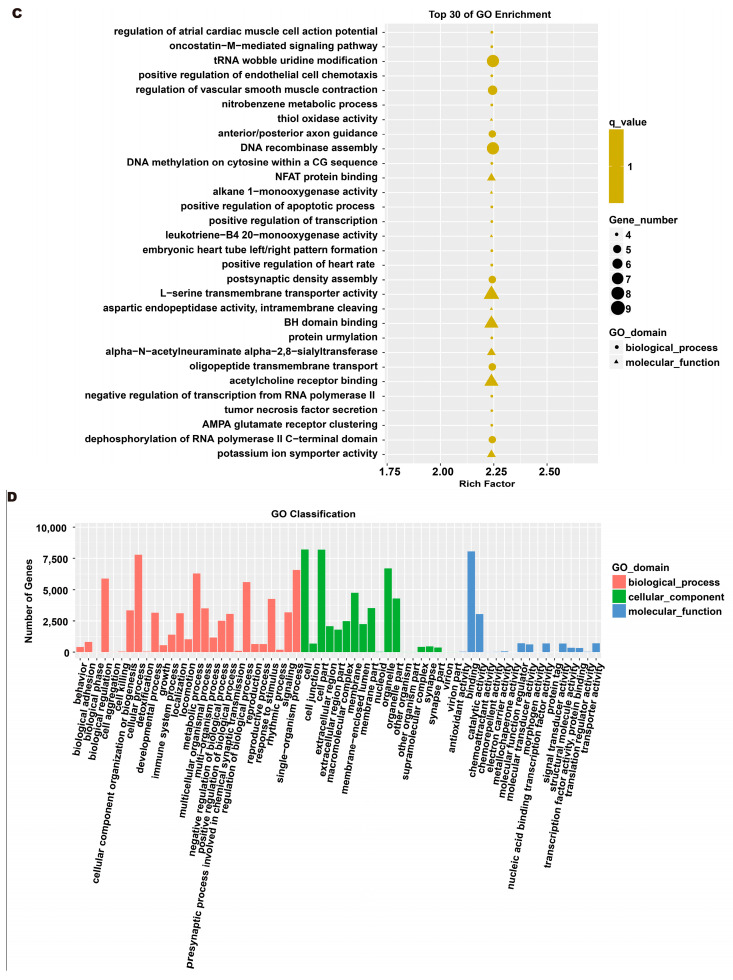
Gene ontology (GO) analysis of differentially expressed miRNA targets. (**A**) GO classification analysis between RSV (128 μM) group and control group. (**B**) The top 30 GO terms for the target genes of differentially expressed miRNA between RSV (128 μM) group and control group. (**C**) GO classification analysis between RSV (256 μM) group and control group. (**D**) The top 30 GO terms for the target genes of differentially expressed miRNA between RSV (256 μM) group and control group.

**Figure 7:**
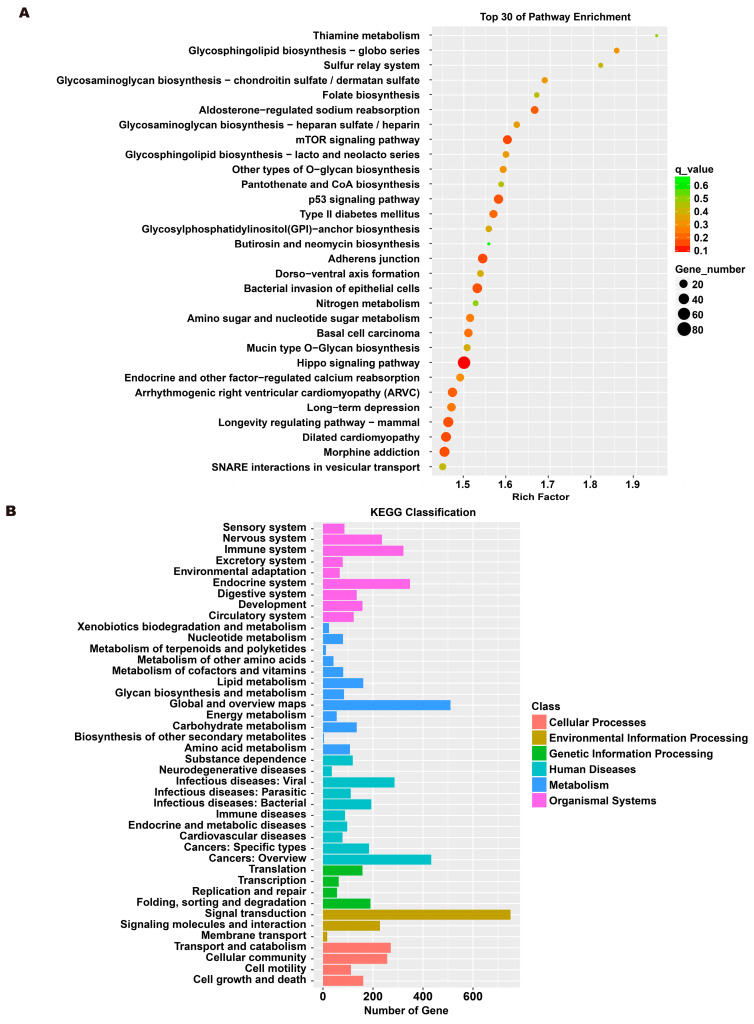

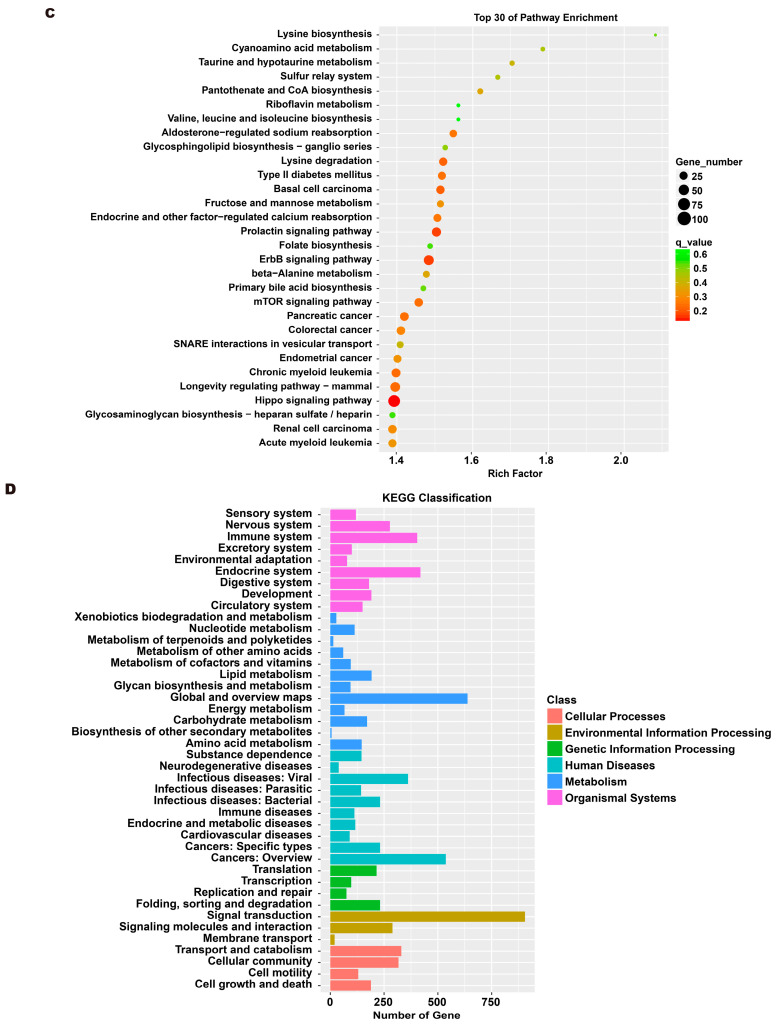
Kyoto encyclopedia of genes and genomes (KEGG) analysis of differentially expressed miRNA targets. (**A**) KEGG classification analysis between RSV (128 μM) group and control group. (**B**) The top 30 KEGG terms for the target genes of differentially expressed miRNA between RSV (128 μM) group and control group. (**C**) KEGG classification analysis between RSV (256 μM) group and control group. (**D**) The top 30 KEGG terms for the target genes of differentially expressed miRNA between RSV (256 μM) group and control group.

To investigate the molecular mechanism through which exosomes activate SH-SY5Y cells, an analysis of exosomes’ miRNA expression profile was conducted. In the treatment group and the control group, 26 miRNAs showed differential expression with 11 up-regulated and 15 down-regulated. In the group treated with 256 μM RSV, 25 miRNAs showed differential expression compared to the control group. Out of these, 21 miRNAs were up-regulated while 4 miRNAs were down-regulated (Figure 5). The target genes of these different miRNAs were predicted using TargetScan, and an analysis of GO enrichment was performed on them. Target genes primarily pertain to cellular processes, metabolic processes, and biological regulation in the context of biological process classification. Cell membranes and organelles are the primary focus of the target genes when considering cell components. Approximately half of the genes in the molecular function study exhibited antioxidant activity, while the majority of genes were engaged in catalysis (Figure 6A,C). Based on pathway categorization, there was a notable enrichment of both dynactin complexes and the binding of transcription coactivators (Figure 6B,D). miRPath was used to analyze the miRNA target genes and identify KEGG-enriched biological pathways. The majority of the target genes of the selected 30 miRNAs were involved in the AMPK signaling pathway, cholinergic synapse, phospholipase D signaling pathway, and ras signaling pathway (Figure 7A–D). The majority of the target genes of the selected 14 miRNAs were involved in ‘Parkinson’s disease’, ‘Alzheimer’s disease’, ‘function in synaptic vesicle exocytosis’, ‘phospholipase D signaling pathway’, ‘glutathione metabolism’, and ‘axon guidance’ (Table 1). The results showed that miRNAs in exosomes obtained from RSV-treated Caco-2 cells activate neuronal cells through their target genes.

**Figure 8:**
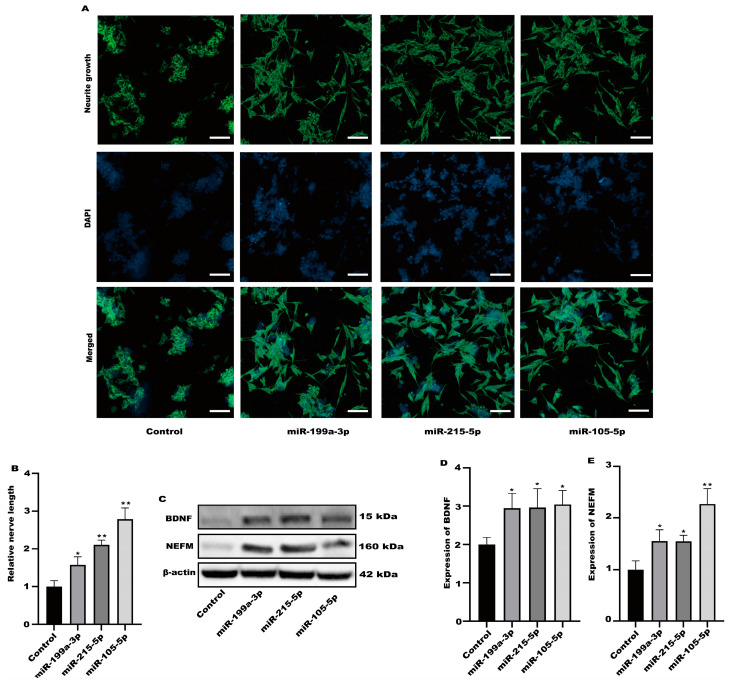
The miRNA in exosomes could activate SH-SY5Y cells. (**A**) Representative fluorescence images of SH-SY5Y were taken with a confocal laser microscope, scale bars: 100 µm. (**B**) Neurofilament medium polypeptide was quantitatively determined via ImageJ version 1.54. (**C**–**E**) The expression of BDNF and NEFM were measured. Values are presented as the means ± SD where applicable (n = 6). * *p* < 0.05 was considered to indicate a statistically significant difference (* *p* < 0.05; ** *p* < 0.01).

Among the altered miRNAs, the focus was on miR-199a-3p, miR-215-5p, and miR-105-5p. These miRNAs are associated with neuronal activation. We evaluated the activation of SH-SY5Y cells after treatment with miR-199a-3p, miR-215-5p, and miR-105-5p mimics. Compared with the control group, all three simulants activated SH-SY5Y cells (Figure 8A,B). At the same time, the protein expression in SH-SY5Y cells was significantly increased after treatment with three simulants (Figure 8C–E). These data indicate that the miRNA in the exosomes derived from Caco-2 is partially involved in the activation of nerve cells.

## 4. Discussion

Some food ingredients have been shown to affect brain cognitive function, including curcumin, retinoids, and carnosine [17,18]. RSV is a dietary compound with chemo-preventative activity. Studies showed that RSV has effective pharmacological activity and the potential to treat a variety of diseases, including inflammation, diabetes, cardiovascular disease, neurodegeneration, tumors, and cancers [19,20,21,22,23,24]. Many aspects of the molecular mechanism for food-induced gut–brain interactions have been studied, including the direct delivery of food components to the brain. In addition, there are the secretion factors of intestinal cells activated by food ingredients and the delivery of immune cells produced from intestinal cells activated by food ingredients. In addition to the studied direct effects of RSV on the brain, RSV may play a protective role through indirect effects. Therefore, we hypothesized that RSV may activate intestinal cells to secrete factors and induce intestinal–brain interactions.

Exosomes are a concern for many scientific researchers. In particular, food-induced exosomes have been considered for the treatment of diseases [25]. Studies showed that both imidazole dipeptide carnosine and γ-Aminobutyric acid can induce the secretion of exosomes, thereby activating the axon extension in SH-SY5Y cells [26,27]. Our study focused on the exosomes secreted by RSV-treated Caco-2 cells, which can induce neurite extension in SH-SY5Y cells. First, SH-SY5Y cell neurites grow more rapidly in a Caco-2 medium containing RSV. Second, Caco-2 cells were cultured in a Transwell polycarbonate membrane, and the collected fluid can also promote SH-SY5Y neurite growth. Finally, TEM was used to identify the collected exosomes, which can also be used to promote neurite growth in SH-SY5Y cells. Compared with RSV-stimulated Caco-2 cell culture medium, 400 nm Transwell polycarbonate membrane blocks the passage of organic macromolecules larger than 400 nm. The Transwell polycarbonate membrane only allows the passage of exosomes and small molecules smaller than 400 nm, allowing researchers to further focus on the experimental subject. The exosome collection kit was used to further confirm that exosomes smaller than 400 nm play a role in activating nerve cells. The innovation of this study is to focus on the exosomes produced by RSV-stimulated Caco-2 cells according to normal cell culture medium, Transwell polycarbonate membrane filtered cell culture medium, and an exosome collection kit, and to detect miRNA in exosomes. Overall, the results indicated that RSV can induce the secretion of exosomes from intestinal cells. Exosomes can play a role by transporting exosomes to the central nervous system through the bloodstream, penetrating the blood–brain barrier, and activating nerve cells in the brain. In their seminal study, Alvarez Erviti et al. pioneered the utilization of exosomes as a vehicle for gene transfer [28]. Specifically, they employed brain-targeted exosomes to administer BACE1 siRNA, with the aim of addressing Alzheimer’s disease. Remarkably, these extracellular vesicles not only exhibited commendable efficacy in delivering the therapeutic cargo to cells in vitro, but also elicited a substantial reduction in both BACE1 mRNA and protein levels upon intravenous administration, primarily within the midbrain, cortex, and striatum. The capacity of extracellular vesicles to traverse the blood–brain barrier may be intricately linked to the process of RVG-mediated endocytosis. Additionally, research suggests that exosomes have the ability to internalize within multivesicular bodies and subsequently rerelease, thereby exerting their effects [29]. This phenomenon could potentially serve as an alternative mechanism through which extracellular vesicles traverse multiple layers of the blood–brain barrier. However, further evidence is necessary to fully elucidate the precise mechanism. In this study, RSV was able to induce the production of exosomes in Caco-2 cells. Since exosomes can cross the blood–brain barrier to exert their effects. We have reason to suspect that certain substances in exosomes may play a positive role in activating neurons. This study can provide a novel insight into the treatment of neurological diseases. So, we need to conduct detailed research on exosomes. We used second-generation sequencing methods to sequence miRNAs in exosomes. RSV-induced Caco-2 cells produced miRNA and exosomes that were analyzed for expression profiles. Compared with the control group, the expression of miRNA in the RSV treatment group changed significantly. Target genes of differentially expressed miRNAs were enriched via GO and KEGG, and were mainly enriched in multiple signaling pathways, such as the AMPK signaling pathway, cholinergic synapse, phospholipase D signaling pathway, and ras signaling pathway. We selected several differentially expressed miRNAs (miR-199a-3p, miR-215-5p, and miR-105-5p) according to the literature [30,31,32]. We used three miRNA mimics to intervene in SH-SY5Y cells and found that the three miRNAs can activate SH-SY5Y cells. The above data indicate that RSV can induce Caco-2 cells to produce exosomes. These exosomes contain some specific miRNAs that can activate SH-SY5Y nerve cells. Collectively, the aforementioned studies furnish compelling theoretical substantiation for the auspicious prospects of extracellular vesicles in facilitating gene transmission within the brain.

## 5. Conclusions

**Figure 9:**
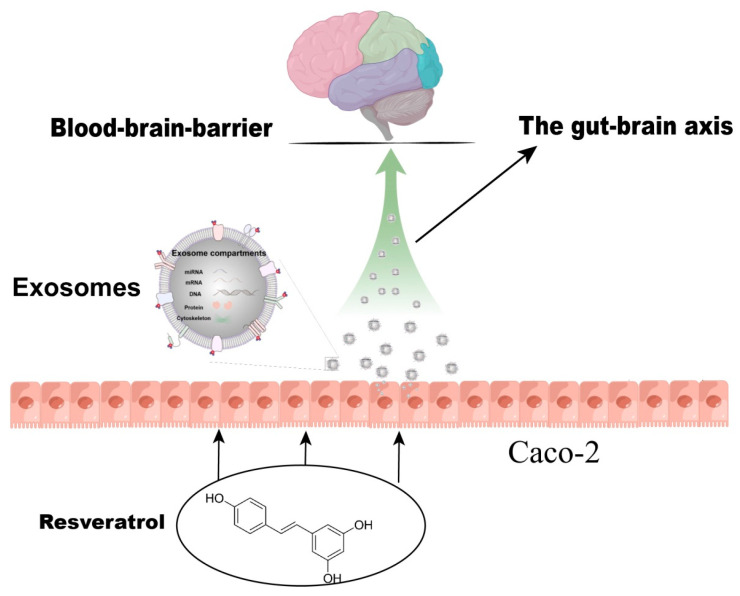
Resveratrol induces neuronal synapse growth by inducing Caco-2 cells to produce exosomes.

RSV-activated Caco-2 cells induced activation of SH-SY5Y cells via the release of exosomes. The miRNA expression profile of exosomes after RSV treatment changed, and 26 kinds of miRNAs expressed differentially between the control group and the RSV-treated group. The miRNAs in the exosomes of RSV-treated intestinal cells may play a key role in the activation of neuronal cells (Figure 9).

## Figures and Tables

**Table 1:** Functions of target genes of miRNA in exosomes derived from RSV-treated Caco-2 cells.

miRNA	Function of Target Genes
miR-122-5p	Transduction of nerve signals
miR-199a-3p	Parkinson’s disease
miR-199b-3p	Role in development of nervous system
miR-320c	Neurodegeneration
miR-215-5p	The neurexin family
miR-146b-3p	AMPK signaling pathway
miR-199a-5p	Ras signaling pathway
miR-432-5p	Function in synaptic vesicle exocytosis
miR-105-5p	Neurodegenerative disorder
miR-199b-5p	Alzheimer’s disease
miR-411-5p	Axon guidance
miR-483-3p	Phospholipase D signaling pathway
miR-6808-3p	Cholinergic synapse

## Data Availability

The data used to support the findings of this study are included within the article.

## Abbreviations

RSV	Resveratrol
TEM	Transmission electron microscopy
AD	Alzheimer’s disease
FBS	Fetal bovine serum
NEAA	Nonessential amino acids
MEM	Modified Eagle Medium
NEFM	Neurofilament medium polypeptide
BDNF	Brain-derived neurotrophic factor
RNA-seq	RNA sequencing
GO	Gene ontology
KEGG	Kyoto encyclopedia of genes and genomes
